# Recognition of Ellipsoid-like Herbaceous Tibetan Medicinal Materials Using DenseNet with Attention and ILBP-Encoded Gabor Features †

**DOI:** 10.3390/e25060847

**Published:** 2023-05-25

**Authors:** Liyuan Zhou, Hongmei Gao, Dingguo Gao, Qijun Zhao

**Affiliations:** School of Information Science and Technology, Tibet University, Lhasa 850011, China; zliyuan@utibet.edu.cn (L.Z.);

**Keywords:** Tibetan medicinal materials, local binary patterns, multi-feature fusion, image recognition

## Abstract

Tibetan medicinal materials play a significant role in Tibetan culture. However, some types of Tibetan medicinal materials share similar shapes and colors, but possess different medicinal properties and functions. The incorrect use of such medicinal materials may lead to poisoning, delayed treatment, and potentially severe consequences for patients. Historically, the identification of ellipsoid-like herbaceous Tibetan medicinal materials has relied on manual identification methods, including observation, touching, tasting, and nasal smell, which heavily rely on the technicians’ accumulated experience and are prone to errors. In this paper, we propose an image-recognition method for ellipsoid-like herbaceous Tibetan medicinal materials that combines texture feature extraction and a deep-learning network. We created an image dataset consisting of 3200 images of 18 types of ellipsoid-like Tibetan medicinal materials. Due to the complex background and high similarity in the shape and color of the ellipsoid-like herbaceous Tibetan medicinal materials in the images, we conducted a multi-feature fusion experiment on the shape, color, and texture features of these materials. To leverage the importance of texture features, we utilized an improved LBP (local binary pattern) algorithm to encode the texture features extracted by the Gabor algorithm. We inputted the final features into the DenseNet network to recognize the images of the ellipsoid-like herbaceous Tibetan medicinal materials. Our approach focuses on extracting important texture information while ignoring irrelevant information such as background clutter to eliminate interference and improve recognition performance. The experimental results show that our proposed method achieved a recognition accuracy of 93.67% on the original dataset and 95.11% on the augmented dataset. In conclusion, our proposed method could aid in the identification and authentication of ellipsoid-like herbaceous Tibetan medicinal materials, reducing errors and ensuring the safe use of Tibetan medicinal materials in healthcare.

## 1. Introduction

**Figure 1:**
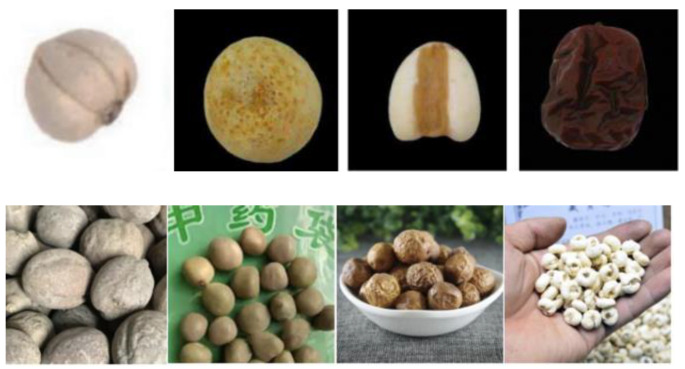
Target images taken against an ideal background and complex backgrounds.

As the material basis of medical theory, Tibetan medicinal materials serve to achieve the purposes of disease prevention and healthcare, acting as a bridge between medical theory and clinical practice [1]. The correct recognition and application of Tibetan medicines are essential prerequisites for making full use of their medicinal value. Ellipsoid-like herbaceous Tibetan medicinal materials have fewer intraclass differences due to their similar natural attributes, such as their color and shape. In the early days, people mainly relied on manual methods of identification, such as observation, touch, taste, and smell, to recognize Tibetan medicinal materials [2]. However, these methods are highly subjective, labor-intensive, and prone to errors. With the development of deep-learning technology, great progress has been made in the recognition of ellipsoid-like herbaceous Tibetan medicinal materials [3,4]. Compared to traditional manual methods, deep-learning-based methods are more efficient at extracting the hidden features and structured information of ellipsoid-like herbaceous Tibetan medicinal materials. They overcome subjective human influences. Although deep-learning-based methods have made some progress in identifying Chinese herbs with similar shapes and colors, recognizing Tibetan medicinal materials still poses many challenges. Firstly, the existing recognition methods mainly focus on medicinal material images with a single target and a simple background in ideal laboratory environments (as shown in Figure 1), which perform poorly in realistic environments with multiple targets and a complex background. Secondly, under a complex background, the underlying features of an image easily change with a change in the background [5], and cannot be leveraged as a reliable recognition feature.

To address these challenges, we built a standard dataset of ellipsoid-like herbaceous Tibetan medicinal materials with complex backgrounds. We combined a Gabor wavelet transform and improved local binary patterns to extract the texture features of images, and used the DenseNet network with an added attention mechanism to identify ellipsoid-like herbaceous Tibetan medicine images. The experimental results show that our method can achieve a 93.67% recognition accuracy on our dataset. To sum up, the main contributions of this paper are as follows:

- We verified the key role of texture features in recognizing ellipsoid-like herbaceous Tibetan medicinal materials by conducting multi-feature fusion experiments on a constructed ellipsoid-like herbaceous Tibetan medicinal material dataset.
- We used data enhancement to increase the number of images and validated its effectiveness in the recognition of ellipsoid-like herbaceous Tibetan medicinal materials.
- We proposed the use of an improved LBP algorithm to encode texture features of ellipsoid-like herbaceous Tibetan medicinal materials and demonstrated its effectiveness at improving the recognition accuracy on an additional complex test set.
- We evaluated our proposed method against existing herbal methods on the constructed dataset, and our results show that our method achieved better recognition for ellipsoid-like herbaceous Tibetan medicinal materials on a complex background.

The remainder of this paper is organized as follows: Section 2 reviews related work, Section 3 presents the method for identifying ellipsoid-like herbaceous Tibetan medicinal materials, Section 4 presents the experiments and an analysis of the results, and Section 5 concludes the paper.

## 2. Related Work

Achievements have been made in the computer-based recognition of herbal medicines with similar shapes. Earlier works have relied on the underlying features of a single image, such as the color [6,7,8,9], texture [10,11,12,13,14], and shape [5,15], for various fine-grained herbal medicine recognition tasks. Due to the richness of herbal species, even herbs belonging to the same species can vary significantly in quality due to differences in the growing regions, climate, harvesting times, and processing methods. Recent research has proposed the use of deep-learning networks in the field of traditional Chinese medicine recognition, with convolutional neural networks showing greater advantages over traditional shallow machine-learning algorithms in image classification. The main deep-learning algorithms used in this field include GoogleNet [16,17,18,19], VGGNet [16,17,18,20,21], ResNet [20,22,23], DenseNet [24], and AlexNet [20,21,25,26,27,28], among others. Lightweight CNNs such as SqueezeNet [29], ShuffleNet [30,31], and MobileNet [32,33,34] are also gaining popularity due to their fast speed, small memory requirement, and low computation, making them suitable for mobile devices. Recent advancements in peripheral vision [35], multi-axis vision transformers [36], and visual transformers [37,38,39] have also improved the accuracy of fine-grained classification tasks. These methods provide important references for recognizing ellipsoid-like herbaceous Tibetan medicinal materials. However, existing experiments have mainly focused on images of individual ellipsoid-like herbaceous Tibetan medicinal materials taken in ideal environments, resulting in degraded recognition effects for images with complex backgrounds. In this paper, we propose a recognition model that combines texture feature extraction with deep-learning methods for images of ellipsoid-like herbaceous Tibetan medicinal materials captured in complex backgrounds. We improved the model’s robustness to complex background distractors by introducing an attention mechanism.

## 3. Materials and Methods

### 3.1. A Dataset of Ellipsoid-like Herbaceous Tibetan Medicinal Materials

**Figure 2:**
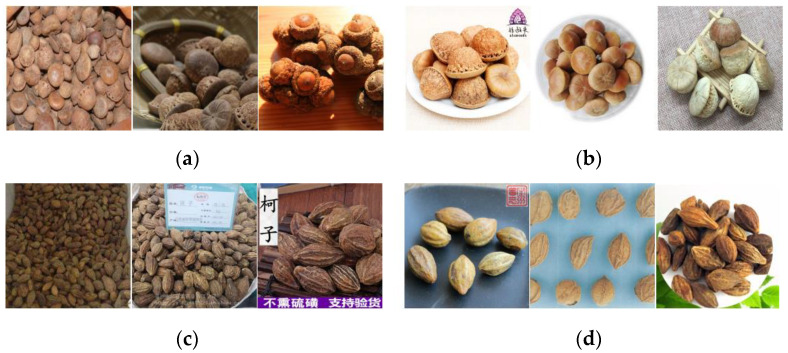
Images in the complex test set and the normal test set. (**a**) Complex test set. (**b**) Test set. (**c**) Complex test set. (**d**) Test set.

By reviewing the Encyclopedia of Tibetan Medicinal Materials in China, we selected 18 types of ellipsoid-like herbaceous Tibetan medicinal materials, including Lu Lu Tong, soapberry, and You Ma Zhong. We leveraged Python 3.8 [40] to search for corresponding images of ellipsoid-like herbaceous Tibetan medicinal materials from the Bing search engine and major Tibetan medicinal material websites. Additionally, we went to the Tibetan Museum of Nature Sciences to take some pictures of ellipsoid-like herbaceous Tibetan medicinal materials. Due to the low quality of the captured images, manual screening was required to ensure that the images in the original dataset correctly reflected the corresponding ellipsoid-like herbaceous Tibetan medicinal materials. Therefore, we hired researchers specializing in Tibetan medicine to identify and screen the images in the dataset, ensuring their accuracy. In total, we acquired 3200 images of 18 species of ellipsoid-like herbaceous Tibetan medicinal materials. After the manual screening, many images in the dataset that were obtained from the internet were discarded for some types of ellipsoid-like herbaceous Tibetan medicinal materials. Meanwhile, the number of images obtained by field photography at Lhasa joint specialty stores and the Tibetan Museum of Natural Sciences was also limited. As a result, the available training images for model learning were insufficient. To address this issue, we used data augmentation methods to expand the dataset by adjusting the brightness, adding Gaussian noise, and mirroring and rotating the images. In this way, the training dataset was enlarged and the number of Tibetan medicinal material images increased from 3200 to 16,000, which helped alleviate the model overfitting issue [4]. To evaluate the effect of data augmentation on the recognition of ellipsoid-like herbaceous Tibetan medicinal materials, we conducted experiments on the original dataset and the augmented dataset using our proposed method. The dataset was randomly divided into a training set and a test set in an 8:2 ratio. To evaluate the recognition accuracy of our proposed model, we additionally collected 360 images of complex backgrounds to build a complex test set. Compared with the images in the normal test set, the images in the complex test set had backgrounds that were usually very similar in color to the medicinal materials, and the occlusion was more severe. Some example images are shown in Figure 2.

### 3.2. Multi-Feature Fusion

#### 3.2.1. Feature Extraction

The color features of the images were less sensitive to size and orientation and had clear, intuitive, and easily described physical properties [41]. The RGB (red, green, blue) encoding method was used to represent the intensity of each of the three color channels: red, green, and blue. After encoding, the RGB color space was converted to the HSI (hue, saturation, intensity) color space for extracting the image feature vectors, and the obtained color feature vectors were subsequently normalized. Compared with the RGB model, the HSI model adds two feature parameters: saturation and luminance. Assuming that the values of the color components in the RGB color space are $\left(R,G,B\right)$ and $\left(R,G,B\right)\in \left[0,1\right]$ the formulas for converting from the RGB color space to the HSI color space are as follows [42]:

(1) $$H=\left\{\begin{array}{c} \theta ,B\leq G\;\;\;\;\;\;\;\;\;\;\\ 2\pi -\theta ,B>G \end{array}\right.$$

(2) $$S=1-\frac{3\min\left(R,G,B\right)}{R+G+B}$$

(3) $$I=\frac{1}{3}\left(R+G+B\right)$$

where $\theta =\mathrm{arc}\cos\frac{\frac{1}{2}\left[\left(R-G\right)+\left(R-B\right)\right]}{\sqrt{{\left(R-G\right)}^{2}+\left(R-B\right)\left(G-B\right)}}$.

The histogram of oriented gradient (HOG) algorithm is widely used for the shape feature extraction of images. The image is first normalized, and then gamma compression is applied to the color image to reduce the effects of shadows and illumination variations. Then, the gradient calculation is performed on the normalized color image to obtain the horizontal and vertical gradient components, $G_{x}$ and $G_{y}$, and to calculate the current pixel gradient amplitude G. The calculation formula is as follows [43]:

(4) $$\left\{\begin{array}{c} G_{x}=max\left(G_{rx},G_{gx},G_{bx}\right)\\ G_{y}=max\left(G_{ry},G_{gy},G_{by}\right)\\ G_{x,y}=\sqrt{G_{x}{}^{2}+G_{y}{}^{2}}\;\;\;\;\;\;\;\;\;\;\;\; \end{array}\right.$$

The local binary pattern (LBP) texture analysis operator was first proposed by Ojala et al. [44]. This algorithm is widely used in the feature extraction process of recognizing objects [45]. The texture structure characteristics of ellipsoid-like herbaceous Tibetan medicinal materials under different angles and levels of illumination and shading do not change significantly. The local binary model can ideally extract the texture features of ellipsoid-like herbaceous Tibetan medicinal materials, which increases the robustness and accuracy of ellipsoid-like herbaceous Tibetan medicinal material recognition. The LBP algorithm [46] we used is defined as follows:

(5) $$LBP_{P,R}=\sum_{P=0}^{P-1}s\left(g_{p}-g_{c}\right)2^{p},\;s\left(x\right)=\left\{\begin{array}{c} 1,x\geq 0\\ 0,x<0 \end{array}\right.$$

where  P and R represent the number of domain pixels and the processing radius of the processing unit, respectively. $g_{c}$ represents the gray value of the center pixel and $g_{p}$ represents the gray value of the first few pixels in the field,  where P=0, 1, 2, 3….

#### 3.2.2. Multi-Feature Fusion of Images

Ellipsoid-like herbaceous Tibetan medicinal materials have a high similarity in terms of shape and color. Conducting multi-feature fusion experiments on the extracted features can verify the importance of color, shape, and texture features in image representation. We allocated different weights to different features for feature fusion [47]. The total weight of the fused features was 1. Through experiments, we obtained the optimal weights for each feature. The multi-feature fusion equation is as follows:

(6) $$F=aF_{RGB}+bF_{HOG}+cF_{LBP}$$

where F represents the fused features; $F_{RGB}$ represents the color features; $F_{HOG}$ represents the shape features; $F_{LBP}$ represents the texture features; and *a*, *b*, and *c* represent the weight coefficients of each feature, respectively.

#### 3.2.3. ILBP-Encoded Gabor Features

Improved local binary patterns: The basic LBP operator assigns the gray value of all pixels smaller than the central gray value to 0 when extracting the image texture [42]. It does not take into account pixels with small differences from the central gray value, so some useful texture information will be lost. For example, if the center pixel is 90 and the surrounding pixels have a gray value of 89, then obviously assigning the gray value of this surrounding pixel to 0 will result in some loss of information.

In addition, comparing only the grayscale values of the peripheral pixels with the central grayscale value suffers from the influence of the central grayscale value and is not very stable [48]. Therefore, we proposed an improved LBP algorithm. Suppose P stands for the current pixel, the gray value of the current pixel is defined as $g_{r}$, the average gray value of the 8 neighborhoods is $g_{a}$, and the standard deviation of the 8 neighborhoods is $g_{\delta }$. The ILBP (improved local binary pattern) algorithm operator is defined as follows:

(7) $$\left\{\begin{array}{c} P=1,g_{r}\geq g_{a}\;or\;g_{r}<g_{a}\;\mathrm{and}\;g_{a}-g_{r}<g_{\delta }\\ P=0,\;g_{r}<g_{a}\;\mathrm{and}\;g_{a}-g_{r}\geq g_{\delta }\;\;\;\;\;\;\;\;\;\;\;\;\;\;\;\;\;\;\;\;\;\; \end{array}\;\;\;\;\;\;\;\;\;\;\;\;\;\;\right.$$

The improved LBP algorithm, which uses the gray average of 8 neighborhoods instead of the central gray value when calculating the binary sequence and considers the variance of the neighborhoods, reduces the influence of the central gray value on the LBP operator and can extract the texture features of the image more effectively.

The process of ILBP-encoded Gabor feature extraction is as follows: the Gabor wavelet can reduce the interference of external factors and extract feature information from multiple angles and scales of the target image, while the LBP algorithm can better present the local feature information of the image, extract clearer local texture features, and reduce the feature dimension of the image [49]. Combining different algorithms can make up for the deficiencies between the other algorithms to a certain extent. The specific implementation process of the algorithm combination can be seen in Algorithm 1.
**Algorithm 1:** ILBP encoded Gabor feature Algorithm.
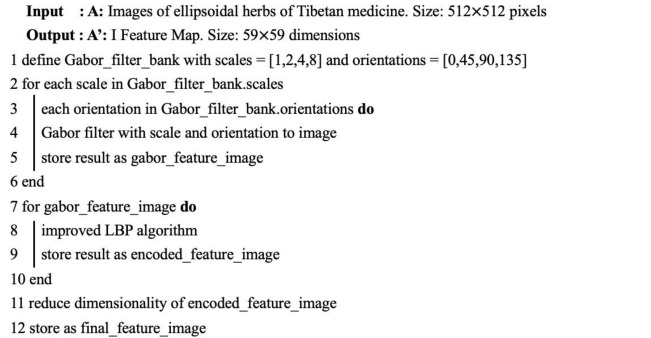



### 3.3. Attentional Mechanisms

The attention mechanism was originally inspired by the human brain’s signal-processing mechanism for vision. When the brain receives information from the external world, it selectively processes only the important information while filtering out the distracting information, thus enhancing the efficiency of information processing [50]. In cognitive science, humans are known to selectively focus on a portion of all information when faced with a large and complex scene, such as regions of abrupt color or style changes, while ignoring other relatively mundane regions due to bottlenecks in information processing. The attention mechanism in computer vision draws from this concept, allowing the network to focus on the important information and ignore the unimportant information. Its first application was in natural language processing, and it was later extended to image processing. Since images of ellipsoid-like herbaceous Tibetan medicinal materials with complex backgrounds often contain irrelevant information, the recognition of these images is usually based on the texture features of the slices that occupy only a part of the image. In this paper, we introduced the attention mechanism into the DenseNet network to focus on the key areas of texture features of ellipsoid-like herbaceous Tibetan medicinal material images with complex backgrounds and extract more accurate key texture feature information, thus enhancing the recognition accuracy.

### 3.4. Construction of a Recognition Model for Ellipsoid-like Herbaceous Tibetan Medicinal Materials

**Figure 3:**
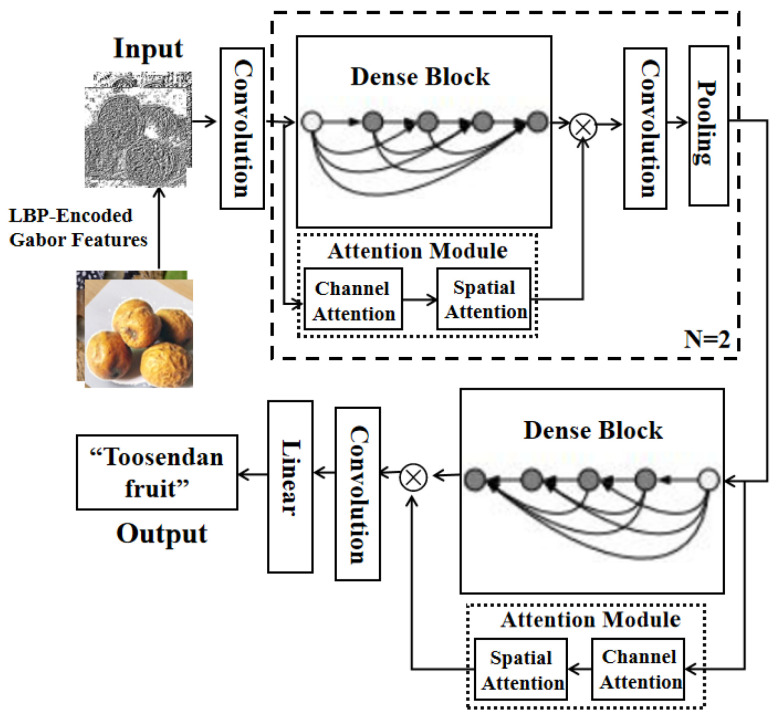
DenseNet with attention and LBP-encoded Gabor features model.

To construct a recognition model for ellipsoid-like herbaceous Tibetan medicinal materials, we used the DenseNet proposed by Huang et al. [51] in 2017 as the backbone network. Due to the uneven distribution of the collected ellipsoid-like herbaceous Tibetan medicinal materials, we changed the loss function to focal loss, which can eliminate the category imbalance and mine difficult samples, improving the image recognition accuracy of the DenseNet network. The complex background of spherical herbaceous Tibetan medicine images may contain invalid information such as utensils and human hands, which may affect the accuracy of a quality evaluation during the training process. Therefore, when training recognition models, it is crucial to introduce an attention mechanism that preserves the target object’s location features while removing background features [52]. First, through a series of convolutions and downsampling, high-level features were gradually extracted to increase the receptive field of the model. Activation pixels in high-level features can reflect regions of interest. Then, the same amount of upsampling was achieved by bilinear differencing to upscale the attention map to the same size as the original input. In this way, an attention region corresponded to each input pixel to obtain an attention map. The channel attention mechanism was introduced to the feature maps of different scales used to generate candidate regions. Instead of considering the feature information of all channels in the feature maps equally, we assigned different weights to each channel of the feature maps. For each channel, we increased the weights of object regions and decreased the weights of non-object regions by weight adaptation. Therefore, the model focused on the valid information with large weights while mitigating the interference of background information. To summarize, based on the spatial attention map, the feature map of each channel was multiplied by the corresponding weight to achieve the final attention mechanism. The attention mechanism unit introduced in each dense block structure can strengthen the global features in the shallow network and re-weight the important channels of each feature in the deep network, thus enhancing the model’s accuracy. Finally, we formed the DenseNet with attention and ILBP-encoded Gabor features. The designed network structure is shown in Figure 3. 

## 4. Results

### 4.1. Experimental Settings

To verify the effectiveness of our method, we conducted experiments on the dataset of ellipsoid-like herbaceous Tibetan medicinal material images with complex backgrounds. We first inputted the image features into the DenseNet network with the attention mechanism after multi-feature fusion, and obtained the optimal feature weights for different features in multi-feature fusion by experimentally comparing the recognition accuracy of images with different weights. We then verified the performance of the network when using a single LBP or Gabor algorithm, LBP-encoded Gabor, ILBP-encoded Gabor, and an attention mechanism. The accuracy of each model was compared and analyzed. We used the adaptive momentum stochastic optimization algorithm to update the weights and biases in the network model. The parameters in the experiment were set as follows: the network learning rate was set to 0.001 and the batch size was set to 16. In the experiment, the stochastic gradient descent method was used for network training, the number of network iterations (Epoch) was set to 50, and focal loss was used as the loss function. We set the hyperparameters α and γ of the focal loss to 0.25 and 2, respectively. The accuracy rate (accuracy) and macro-F1 were used as the evaluation indexes of the model. 

### 4.2. Experimental Results and Analysis

#### 4.2.1. Multi-Feature Fusion Experiment

Table 1 shows the image recognition accuracy and macro-F1 score under different weight assignments. The weighting factors *a*, *b*, and *c* represent the color, shape, and texture features, respectively. The highest accuracy and macro-F1 score for the recognition of complex background ellipsoid-like herbaceous Tibetan medicinal material images were achieved when *a* = 0.1, *b* = 0.1, and *c* = 0.8. Although the color and shape features played a role in classifying and recognizing different types of ellipsoid-like herbaceous Tibetan medicinal materials, they were easily influenced by background interference. The complexity of the texture structure of these materials made texture characteristics crucial for their identification. The weights of different features indicated that texture features play a key role in expressing the image content information. An analysis of the weights of different features concluded that texture features in ellipsoid-like herbaceous Tibetan medicinal material images play a key role in expressing the content information of the images.

#### 4.2.2. Results and Analysis of Ablation Experiments

**Figure 4:**
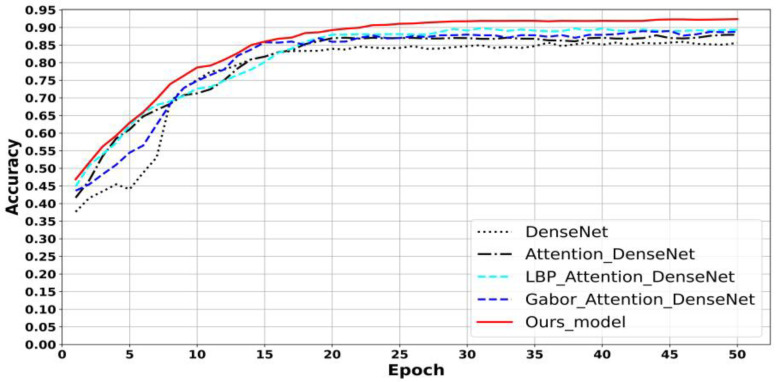
Results of LBP and other methods.

The results of the ablation experiment (shown in Figure 4) demonstrate that the recognition accuracy of the network model gradually improved and eventually stabilized with an increase in the number of iterations in the training process. The recognition accuracy of our model (DenseNet with attention and LBP-encoded Gabor features) was 92.38%, which is higher than that of models using a single LBP or Gabor algorithm. The added attention module improved the model’s feature extraction ability, reducing the weight of useless information and increasing that of useful information. In turn, this improved the overall performance of the network. Texture feature extraction provided a comprehensive understanding of the distinguishing features of ellipsoid-like herbaceous Tibetan medicinal materials, resulting in better classification and recognition results. The ablation experiment results confirmed that using LBP-encoded Gabor resulted in better texture features. This experiment was performed using the original LBP algorithm and the improved LBP (ILBP) method under the same experimental setup, and the results are shown in Table 2. As can be seen, the texture features extracted by the improved LBP operator had a better recognition performance and achieved a 93.67% recognition accuracy. The improved LBP algorithm can extract texture features more effectively by replacing the central gray value with the gray average of the eight neighborhoods and considering the variance of the neighborhoods to reduce the influence of the central gray value on the LBP operator when calculating the binary sequence.

**Figure 5:**
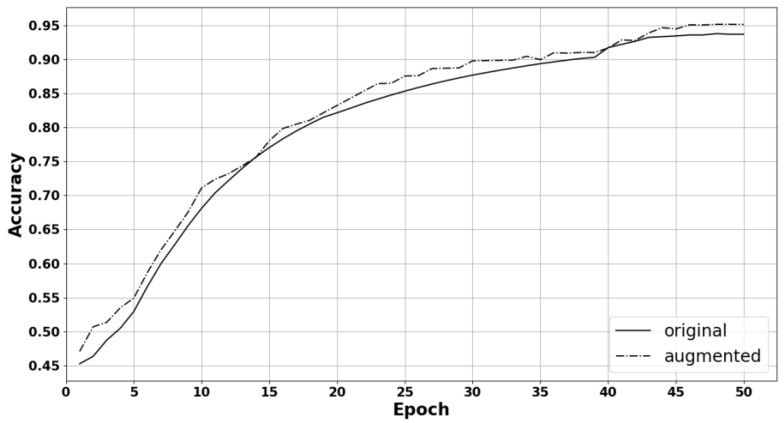
Experimental results for the original and augmented datasets.

To verify the effectiveness of data augmentation, the model was trained using the original data and the data-augmented dataset, and then the experimental results were obtained afterwards using the original data images for testing. The experimental results using our method for the original dataset and the augmented dataset are shown in Figure 5. The recognition accuracy for the original dataset was 93.67%, and the recognition accuracy after performing data augmentation was 95.11%. These results indicate that data augmentation can increase the number of training samples and reduce network overfitting, ultimately improving the model generalization and robustness.

### 4.3. Verification of the Validity of Dilated Convolution

Dilated convolution [53] is able to increase the output cell’s perceptual field without increasing the number of parameters by injecting holes of weight 0 at intervals in the elements inside the conventional convolution kernel, and the number of injected holes is called the dilated rate [54]. Most images of ellipsoid-like herbaceous Tibetan medicinal materials with complex backgrounds contain a lot of invalid scenes, apparatuses, and other information. To alleviate the impact of such distractions, the 3 × 3 convolution in the first dense block was changed into a 3 × 3 convolution of cavities, and the number of cavities in the dilated convolution was two, which can reasonably increase the perceptual field range. For the hole convolution with the expansion rate of 2, the output size was kept constant by setting the step size to 1 and the fill value to 2. The experiments were conducted on the collected complex-background ellipsoid-like herbaceous Tibetan medicinal material image dataset. The experimental results are shown in Table 3. As can be seen, the recognition accuracy after dilated convolution was incorporated into the method in this paper was 89.72%. Compared with the single use of the DenseNet network, the recognition accuracy increased by about 1%, and the recognition accuracy decreased compared with the proposed ellipsoid-like herbaceous Tibetan medicinal material recognition method in this paper (ILBP-encoded Gabor_attention_DenseNet). The reason for this result might be because, although dilated convolution can expand the perceptual field, the convolution results obtained by a certain layer come from an independent set of the previous layer, and there is no correlation between the convolution results of this layer, resulting in the loss of local information.

### 4.4. Comparative Experimental Results and Analysis

In this section, the proposed model is compared with existing methods for identifying traditional Chinese medicines with similar shapes. The model was trained using both the original dataset and the dataset after data augmentation, and then tested using the original dataset images. The experimental results are shown in Table 4. The results indicate that the recognition accuracy and macro-F1 score for the augmented dataset were generally higher than those for the original dataset. Compared with other methods, the method proposed in this paper performed better on both datasets.

The color moment + SVM model [54] had the lowest accuracy among the seven comparison models, as this model extracts color features from images and then classifies them. However, the color features of ellipsoid-like herbaceous Tibetan medicinal materials are highly similar and cannot be used as reliable recognition features. Therefore, on the complex-background dataset of ellipsoid-like herbaceous Tibetan medicinal materials, the color moment + SVM model performed poorly. The existing ResNet [20], Inception-V3 [15], LeNet-5 [54], and YOLOv3 [55] networks achieved recognition accuracies of over 80% on images of ellipsoid-like herbaceous Tibetan medicinal materials on complex backgrounds, but they still had a large gap in their recognition accuracies and macro-F1 scores compared with the method proposed in this paper. YOLOv5-Ghost-CA [56], based on the YOLOv5 algorithm’s backbone network, designed a lightweight GhostBottleneck module. The attention mechanism was added to the model structure and the original convolution layer was replaced with depthwise-separable convolution. This method achieved a recognition accuracy of 89.77% on the dataset in this paper and performed better than other existing traditional Chinese medicine recognition methods. The experimental results demonstrate that the method proposed in this paper had the highest accuracy and the best performance in the comparative experiment.

### 4.5. Experimental Validation on Complex Test Sets

**Figure 6:**
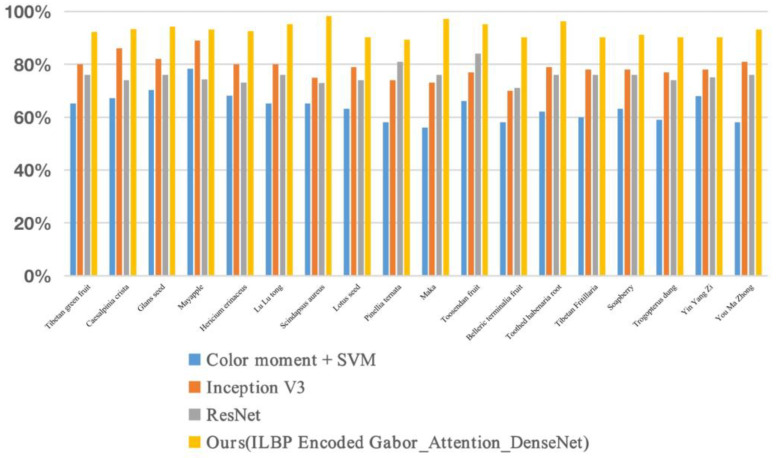
Recognition results of different models on complex test sets.

**Figure 7:**
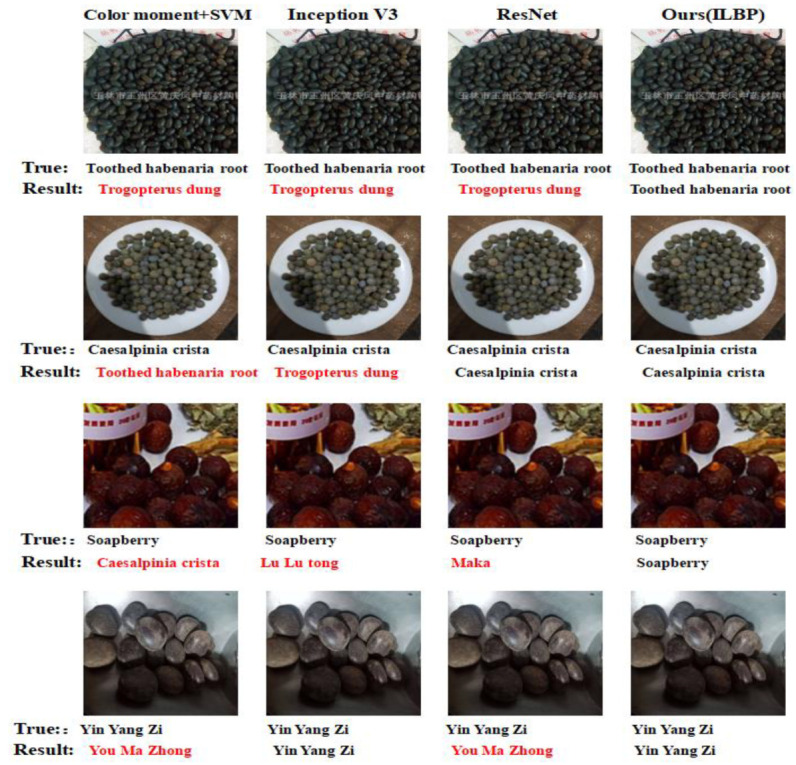
Selected image recognition results of different models on complex test set.

The recognition performance of various models on the complex test set is presented in Figure 6, clearly indicating that the convolutional neural-network-based classification algorithm outperformed traditional shallow machine-learning algorithms in terms of image classification accuracy. Especially in scenarios where the herb color was similar to the background or when the image was heavily occluded, our proposed method achieved a better recognition accuracy than other models, with an average recognition accuracy of 92.41% for 18 types of ellipsoid-like herbaceous Tibetan medicinal materials. The experimental results demonstrate that the combination of traditional texture features (ILBP-encoded Gabor) with deep learning (DenseNet) and the integration of an attention mechanism can effectively improve the recognition accuracy for images with complex backgrounds. Figure 7 illustrates the recognition results of partial images using different methods.

## 5. Discussion

In this paper, based on the established ellipsoid-like herbaceous Tibetan medicinal material dataset, we first verified the criticality of texture features for distinguishing different medicinal material images by multi-feature fusion experiments. We proposed DenseNet models with attention and LBP-encoded Gabor features to recognize ellipsoid-like herbaceous Tibetan medicinal materials on complex backgrounds, and proposed an improved LBP algorithm for texture feature extraction. We discussed the effectiveness of data augmentation for this paper’s research through experiments, and the experimental results prove that data augmentation can effectively improve the recognition accuracy of the experimental results. Our method achieved 93.67% accuracy on the original dataset and 95.11% accuracy on the augmented dataset. We additionally selected images with backgrounds more similar to the medicinal materials as a complex test set, and showed that our proposed method obtained a higher accuracy on this test set compared to other methods. Yet, the secondary recognition of misidentified ellipsoid-like herbaceous Tibetan medicinal materials still needs to be performed manually to ensure the safety of medication. The number of images in the dataset of this experiment was smaller than the standard public dataset CIFAR-10. Although the proposed model has achieved improvements in accuracy, there is still much room for improvement compared with the ideal case of Chinese medicinal material recognition. In the next work, the finished Tibetan medicinal material dataset constructed in this paper will be further expanded and unsupervised or semi-supervised methods will be used to solve the annotation problem of a high-cost, large-scale, ellipsoid-like herbaceous Tibetan medicinal material dataset.

## Figures and Tables

**Table 1:** Different weight assignments and image recognition accuracies.

*a*	*b*	*c*	Accuracy	Macro-F1
0.05	0.05	0.9	89.72%	89.44%
**0.1**	**0.1**	**0.8**	**91.63%**	**91.36%**
0.1	0.15	0.75	90.84%	90.56%
0.15	0.15	0.7	88.86%	88.55%
0.2	0.2	0.6	87.74%	87.31%
0.25	0.25	0.5	85.26%	85.02%
0.3	0.3	0.4	84.68%	84.23%

**Table 2:** Experimental results of the original LBP and the improved local binary patterns (ILBP).

Method	Accuracy	Macro-F1
LBP	92.38%	92.06%
**ILBP**	**93.67%**	**93.65%**

**Table 3:** Experimental results using dilated convolution.

Method	Accuracy	Macro-F1
DenseNet	85.06%	85.05%
DenseNet + dilated	86.23%	86.01%
DenseNet + dilated + attention	87.11%	86.98%
ILBP-encoded Gabor_attention_DenseNet + dilated convolution	89.72%	89.56%
**Ours (ILBPencoded Gabor_attention_DenseNet)**	**93.67%**	**93.65%**

**Table 4:** Different models with different datasets: comparative experimental results.

Method	Dataset	Accuracy	Macro-F1
Color moment + SVM [54]	original dataset	76.43%	76.33%
	augmented dataset	77.75%	77.72%
ResNet [20]	original dataset	82.17%	82.09%
	augmented dataset	86.32%	86.11%
Inception V3 [15]	original dataset	84.06%	83.96%
	augmented dataset	86.64%	86.53%
LeNet-5 [24]	original dataset	80.92%	80.77%
	augmented dataset	82.76%	82.67%
YOLOv3 [55]	original dataset	82.39%	82.24%
	augmented dataset	85.48%	85.40%
YOLOv5-Ghost-CA [56]	original dataset	85.53%	85.28%
	augmented dataset	89.77%	89.70%
**Ours (ILBP-encoded Gabor_attention_DenseNet)**	original dataset	**93.67%**	**93.65%**
	augmented dataset	**95.11%**	**95.09%**

## Data Availability

While we appreciate the potential benefits of sharing our dataset, the sensitive nature of the data prevents us from doing so. The dataset contains data on cherished Tibetan medicinal herbs that cannot be made public without violating biodiversity security. We will be happy to provide access upon request without disclosing the agreement.

## References

[1] Geng Z. (2019). Research on Intellectual Property Legal Protection of Traditional Tibetan Medicine in Tibet.

[2] Shi P., Zheng X. (2020). Application and Prospect of Computer Vision in the Field of Traditional Chinese Medicine. J. Tex. Coll..

[3] Wu C., Tan C., Huang Y., Wu C., Chen H. (2020). Intelligent Identification of Fritillaria, Hawthorn and Pinellia Decoction Pieces Based on Deep Learning Algorithm. Chin. J. Exp..

[4] Zhang Y., Wan H., Tu S. (2022). A Review and Case Study of Computer Vision Based Traditional Chinese Medicine Slice Classification Technology. Comput. Appl..

[5] Zhu L., Li X., Zhang Y., Pu X., Wu C. (2014). Chinese Herbal Medicine Retrieval Method Based on Shape Feature and Texture Feature. Comput. Eng. Des..

[6] Liu B. (2016). Research on Classification Algorithm of Ellipsoid-like Chinese Medicinal Materials Based on Generalized Multi-Kernel Learning.

[7] Li Z. (2013). Traditional Chinese Medicine Slice Feature Extraction and Recognition System.

[8] Yang T. (2014). Research on the “Color Discrimination” of Medicinal Herbs and Decoction Pieces Based on Machine Vision Technology.

[9] Yang T., Lei J., Zhu H., Hu Q.Y., Long B. (2021). Identification of Leaf Diseases in Ophiopogon japonicus Based on Image Feature Fusion. Hubei Agric. Sci..

[10] Tamura H., Moris S., Yamawaki A. (1978). Textural features corresponding to visual perception. IEEE Trans. Syst. Man Cybern..

[11] Cheng M., Zhan Z., Zhang W., Yang H.J., Shen J.Z., Peng H.S. (2019). Textual research of “Huang bo” in classical prescriptions. China J. Chin. Mater. Med..

[12] Yao L., Liang Y. (2000). Identification of Anemarrhena asphodeloides and mixed Anemarrhena asphodeloides. China Pharm..

[13] Tao O., Zhang Y., Chen Q., Wang Y., Qiao Y. (2014). Cross section of traditional Chinese medicine slices based on grayscale co-occurrence matrixExtraction of Image Texture Feature Parameters. World Sci. Technol.—Mod. Tradit. Chin. Med. Hua.

[14] Tao O., Lin Z., Zhang X., Wang Y., Qiao Y. (2014). Texture Feature Parameters Based on Sliced Slice Image Research on the Identification Model of Traditional Chinese Medicine. World Sci. Technol.-Mod. Tradit. Chin. Med..

[15] Hu J., Wang Y., Kan H. (2019). Research on identification of Chinese herbal pieces based on deep transfer learning. J. Xinxiang Univ..

[16] Xu F., Meng S., Wu Q., Lou Z., Chen J., You M., Lu C. (2018). Research on the method of human participation in the identification of American ginseng slices based on convolutional neural networks. J. Nanjing Univ. Tradit. Chin. Med..

[17] Wang J., Dai K., Li Z. (2020). Research on Image Recognition of Traditional Chinese Medicine Slices Based on Deep Learning. Shi Zhen Guoyi Guoyao.

[18] Zhuang Y. (2018). Chinese Herbal Medicine Recognition Based on Deep Neural Networks Guangzhou.

[19] Liu J. (2020). Research on Plant Leaf Recognition Based on Image Analysis.

[20] Fan X., Xu Y., Zhou J., Li Z.L., Peng X., Wang X.T. (2021). Grape leaf disease detection system based on transfer learning and improved. CNN J. Agric. Eng..

[21] Chen Y., Zou L. (2021). Intelligent identification of traditional Chinese medicine decoction pieces based on BMFnet WGAN. Chin. J. Exp. Prescr..

[22] Wang Y., Hao C., Li Y., Chen S. (2020). Micro image recognition of small sample Chinese medicinal powder based on deep learning. Comput. Appl..

[23] Lu K., Xia C., Dai S., Jing H., Ma Y. (2020). Research on the Application of Feature Fusion in Plant Leaf Recognition. Software Guide..

[24] Wu Y., Liu A., Zhu X., Liu C.X., Fan G.H., Le Y., Zhang Y.H. (2021). A convolutional network architecture for plant disease image recognition. J. Anhui Agric. Univ..

[25] Huang F., Yu L., Shen T., Jin L., Xu H., Huang X. (2020). Research and implementation of Chinese herbal medicine plant image classification based on AlexNet deep learning model. J. Qilu Univ. Technol..

[26] Wang Y., Sun W., Zhou X. (2020). Research on Chinese herbal medicine plant image recognition method based on deep learning. Tradit. Chin. Med. Inf..

[27] Zuo Y., Tao Q., Wu L., Wang Y. (2020). Research on Plant Image Classification Method Based on Convolutional Neural Networks. Internet Things Technol..

[28] Sun X., Qian H. (2017). Traditional Chinese Medicine Slice Image Recognition Based on Deep Convolutional Networks. World Sci. Technol.-Mod. Tradit. Chin. Med..

[29] Iandola F., Han S., Moskewicz W., Ashraf K., Dally W.J., Keutzer K. (2016). SqueezeNet: AlexNet-level accuracy with 50x fewer parameters and <0.5 MB model size. arXiv.

[30] Zhang Y., Zhou X., Lin M., Sun J. (2018). ShuffleNet: An extremely efficient convolutional neural network for mobile devices. Proceedings of the 2018 IEEE/CVF Conference on Computer Vision and Pattern Recognition.

[31] Man N., Zhang X., Zheng H., Sun J. (2018). ShuffleNet V2:practical guidelines for efficient CNN architecture design. Proceedings of the 2018 European Conference on ComputerVision, LNCS 11218.

[32] Howard A., Zhu M., Chen B., Kalenichenko D., Wang W., Weyand T., Adam H. (2017). MobileNets: Efficient convolutional neural networks for mobile vision applications. arXiv.

[33] Sandler M., Howard A., Zhu M., Zhmoginov A., Chen L.C. (2018). MobileNetV2: Inverted residuals and linear bottlenecks. Proceedings of the 2018 IEEE/CVF Conference on Computer Vision and Pattern Recognition.

[34] Howard A., Sandler M., Chu G., Chen L.C., Chen B., Tan M., Wang W., Zhu Y., Pang R., Vasudevan V. (2019). Searching for MobileNetV3. Proceedings of the 2019 IEEE/CVF International Conference on Computer Vision.

[35] Mozaffari M.H., Lee W.S. (2020). Semantic Segmentation with Peripheral Vision. ISVC 2020: Advances in Visual Computing.

[36] Tu Z., Talebi H., Zhang H., Yang F., Milanfar P., Bovik A., Li Y., Avidan S., Brostow G., Cissé M., Farinella G.M., Hassner T. (2022). MaxViT: Multi-axis Vision Transformer. Computer Vision—ECCV 2022.

[37] Jiang Y., Chang S., Wang Z. (2021). TransGAN: Two Transformers Can Make One Strong GAN. arXiv.

[38] Yoon D., Oh J., Choi H., Yi M., Kim I. (2022). OUR-GAN: One-shot Ultra-high-Resolution Generative Adversarial Networks. arXiv.

[39] Wu J., Jiang Y., Bai S., Zhang W., Bai X., Avidan S., Brostow G., Cissé M., Farinella G.M., Hassner T. (2022). SeqFormer: Sequential Transformer for Video Instance Segmentation. Computer Vision—ECCV 2022.

[40] Daniel H., Liang E., Stoica I. (2019). Population Based Augmentation: Efficient Learning of Augmentation Policy Schedules. arXiv.

[41] Guo L. (2022). An image research based on color and texture features. Shanxi Electron. Technol..

[42] Lv Y., Wang J. (2019). Image recognition of Chinese herbal pieces based on HOG-LBP features. China J. Tradit. Chin. Med..

[43] Ojala T., Pietikainen M., Maenpaa T. (2002). Multiresolution Gray-Scale and Rotation Invariant Texture Classification with Local Binary Patterns. IEEE Trans. Pattern Anal. Mach. Intell..

[44] Zhang H., Xu S. (2016). The Face Recognition Algorithms Based on Weighted LTP. J. Image Graph..

[45] Yan W., Wang Y., He X., Jiang Y., Wang B., Ji H., Huang Z. (2021). New Image Reconstruction Algorithm for CCERT: LBP + Gaussian Mix Model (GMM) Clustering. Meas. Sci. Technol..

[46] Ji Y., Zhou W. (2021). Fabric image retrieval system based on multi-feature fusion. Comput. Digit. Eng..

[47] Wang G., Zhang P., Ren G., Kou X. (2011). Application of improved local binary model in abrasive image recognition. Lubr. Seal..

[48] Lukas S., Mitra A.R., Desanti R.I., Krisnadi D. (2016). Implementing Discrete Wavelet and Discrete Cosine Transform with Radial Basis Function Neural Network in Facial Image Recognition. J. Image Graph..

[49] Taissir F., Haikel A., Ridha O., Mohamed A. (2020). Electrocardiogram Heartbeat Classification Based on A Deep Convolutional Neural Network and Focal loss. Comput. Biol. Med..

[50] Huang G., Liu Z., Laurens V., Kilian Q. (2017). Densely Connected Convolutional Networks. Proceedings of the 2017 IEEE Conference on Computer Vision and Pattern Recognition.

[51] Wang X., Girshick R., Gupta A., Kaiming H. (2018). Non-local Neural Networks. Proceedings of the IEEE Conference on Computer Vision and Pattern Recognition.

[52] Zhang P., Kong W., Teng J. (2022). Facial expression recognition based on multi-scale feature attention mechanism. Comput. Eng. Appl..

[53] Lai Z., Chen R., Qian Y. (2020). CNN real-time micro-expression recognition algorithm combined with atrous convolution. Comput. Appl. Res..

[54] Zhou T. (2022). Research on Image Recognition of Ethnic Medicine Plants Based on Deep Learning.

[55] Gao S., Zhou Z., Huang X., Gao L., Bian H. (2023). Research on the detection of traditional Chinese medicine tablets based on YOLOv3 algorithm. Chin. Herb. Med..

[56] Dong M., Liang Y., Liu Y., Qi Z., Niu H. (2022). Implementing detection and identification of Chinese medicine tablets based on the improved YOLOv5. Mod. Comput..

